# Mechanisms of constitutive and ATP-evoked ATP release in neonatal mouse olfactory epithelium

**DOI:** 10.1186/1471-2202-13-53

**Published:** 2012-05-28

**Authors:** Sébastien Hayoz, Cuihong Jia, CC Hegg

**Affiliations:** 1 Department of Pharmacology and Toxicology, B439 Life Sciences, Michigan State University, East Lansing, MI, 48824, USA

## Abstract

**Background:**

ATP is an extracellular signaling molecule with many ascribed functions in sensory systems, including the olfactory epithelium. The mechanism(s) by which ATP is released in the olfactory epithelium has not been investigated. Quantitative luciferin-luciferase assays were used to monitor ATP release, and confocal imaging of the fluorescent ATP marker quinacrine was used to monitor ATP release via exocytosis in Swiss Webster mouse neonatal olfactory epithelial slices.

**Results:**

Under control conditions, constitutive release of ATP occurs via exocytosis, hemichannels and ABC transporters and is inhibited by vesicular fusion inhibitor *Clostridium difficile* toxin A and hemichannel and ABC transporter inhibitor probenecid. Constitutive ATP release is negatively regulated by the ATP breakdown product ADP through activation of P2Y receptors, likely via the cAMP/PKA pathway. In vivo studies indicate that constitutive ATP may play a role in neuronal homeostasis as inhibition of exocytosis inhibited normal proliferation in the OE. ATP-evoked ATP release is also present in mouse neonatal OE, triggered by several ionotropic P2X purinergic receptor agonists (ATP, αβMeATP and Bz-ATP) and a G protein-coupled P2Y receptor agonist (UTP). Calcium imaging of P2X_2_-transfected HEK293 “biosensor” cells confirmed the presence of evoked ATP release. Following purinergic receptor stimulation, ATP is released via calcium-dependent exocytosis, activated P2X_1,7_ receptors, activated P2X_7_ receptors that form a complex with pannexin channels, or ABC transporters. The ATP-evoked ATP release is inhibited by the purinergic receptor inhibitor PPADS, *Clostridium difficile* toxin A and two inhibitors of pannexin channels: probenecid and carbenoxolone.

**Conclusions:**

The constitutive release of ATP might be involved in normal cell turn-over or modulation of odorant sensitivity in physiological conditions. Given the growth-promoting effects of ATP, ATP-evoked ATP release following injury could lead to progenitor cell proliferation, differentiation and regeneration. Thus, understanding mechanisms of ATP release is of paramount importance to improve our knowledge about tissue homeostasis and post-injury neuroregeneration. It will lead to development of treatments to restore loss of smell and, when transposed to the central nervous system, improve recovery following central nervous system injury.

## Background

Although once widely assumed that the only source for extracellular ATP was from damaged cells under pathophysiological conditions, it is now accepted that ATP is also released under normal physiological conditions in several organ systems via exocytosis [1], efflux through connexon or pannexin “hemichannels” [2,3], voltage-dependent anion channels [4], and/or activated P2X_7_ receptors [1]. Extracellular ATP has numerous functions in sensory systems. It is released via pannexins to mediate intercellular communication in taste buds [2,3]. In the auditory system, spontaneous ATP release is required for activity in the developing auditory system [5], ATP release through connexin hemichannels propagates calcium signals in the inner ear [6] and purinergic receptor activation by ATP is essential in the cell-cell communication that accompanies cochlear injury [7]. In retinal glial cells, ATP release propagates spontaneous intercellular glial calcium waves that alter the diameter of arterioles in the retina, suggesting released ATP can affect retinal physiology [8].

ATP also mediates essential functions in the olfactory system: (1) ATP activation of purinergic receptors in Swiss Webster mouse olfactory epithelium (OE) induces neuroproliferation [9] (2) ATP has proliferative and protective effects following injury [10,11] and (3) ATP induces upregulation and/or release of various neurotrophic factors [12-14]. Given the multiple roles of ATP in the mouse OE, we hypothesized that ATP was released under both normal and pathophysiological conditions in mouse OE and likely involved multiple mechanisms. Vesicles containing ATP could be released via calcium-dependent exocytosis. Cytosolic ATP could also be released by efflux via ATP binding cassette (ABC) transporters [15,16] expressed in the OE [17]. Several connexin subtypes are expressed in the OE [18-22] and pannexins are expressed in the olfactory bulb [23], but as of yet, no reports have examined pannexin expression in the epithelium. P2X_7_ receptors have been identified in the OE using immunohistochemistry [24]. Thus, there are multiple pathways of ATP release possible in the OE. Determination of ATP release mechanisms is of paramount importance to improve our understanding of the OE regenerative properties.

The aim of this study was first to investigate whether ATP release mechanisms were present in mouse OE, using Swiss Webster neonatal OE slices as a model. Using 3 techniques, we showed that ATP is released in neonatal OE through constitutive and evoked release. We then characterized the mechanisms underlying the two types of ATP release using the luciferin-luciferase assay and the fluorescent ATP marker quinacrine to monitor the loss of ATP fluorescence from endogenous vesicular stores. Our results show that the evoked release of ATP is mediated by purinergic receptor activation and can occur via calcium-dependent exocytosis, efflux of ATP through activated P2X_7_ receptors or activated P2X_7_ receptor/pannexin complexes, and ABC transporters. Confocal calcium imaging of P2X_2_-transfected HEK293 cells used as biosensors further confirmed the presence of the evoked release of ATP. The constitutive release of ATP does not require purinergic receptor activation, but is mediated by continuous exocytosis and efflux through hemichannels or ABC transporters. In vivo studies indicate that constitutive vesicular release may play a role in neuronal homeostasis as inhibition of exocytosis inhibited the normal proliferation in the OE. Overall, determination of the ATP release mechanisms in the OE has identified numerous pharmacological targets to pursue that could modulate neuronal homeostasis.

## Results

### Functional purinergic receptors are present in the neonatal olfactory epithelium

**Figure 1:**
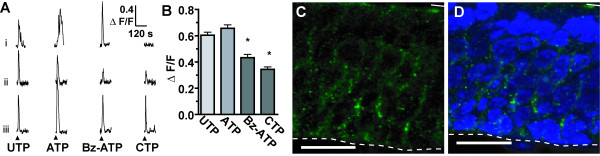
**Functional purinergic receptors expressed in the mouse olfactory epithelium.** (**A**) Representative calcium responses from 3 cells (i-iii) elicited by application (▲) of P2Y agonist UTP (10 μM), P2X and P2Y agonist ATP (25 μM), P2X_1,7_ agonist BzATP (50 μM), or P2X_4_ agonist CTP (100 μM). (**B**) Normalized average peak amplitudes of agonist-elicited calcium responses (mean + SEM). * indicates p < 0.0001 v. ATP group with Bonferroni’s multiple comparison test). **(C-D)** Immunoreactivity of P2X_7_ (green) and DAPI-labeled nuclei (blue). No immunoreactivity was observed with peptide neutralization or omission of primary antibody. Dashed line, basal lamina; solid line, apical surface of OE. Scale bar: 20 μm.

Previous reports using immunohistochemistry indicated that P2X_1,3,4,5,7_ and P2Y_1,2_ receptors are located in the olfactory epithelium [24-26], while P2X_2_ receptors were not located in the OE [26]. Using calcium imaging, we previously showed functional expression of P2Y_1,2_ and P2X_1,3_ receptors [26], but had not examined for the presence of functional P2X_4,5,7_ receptors. To determine the most effective agonists to activate purinergic receptors in our neonatal OE slices preparation, we performed calcium imaging using the selective P2Y_2,4,6_ agonist UTP (10 μM), P2X_4_ agonist cytidine 5’-triphosphate (CTP, 100 μM), P2X_1,2/3,3_ agonist α,β-Methylene ATP (αβMeATP, 10 μM) and P2X_1,7_ agonist 2'(3')-O-(4-Benzoylbenzoyl)ATP (Bz-ATP, 50 μM) (Figure 1A and B). For UTP, CTP and Bz-ATP, we recorded from 230 cells located in the apical OE, presumably from sustentacular cell somas. Of the 230 ATP-responsive cells, 99% (228 cells) responded to UTP, 79% (181 cells) to Bz-ATP, and 36% (83 cells) to CTP. The following agonist profile was observed: ATP = UTP > Bz-ATP = CTP (0.66 ± 0.40, 0.60 ± 0.36, 0.43 ± 0.33, and 0.34 ± 0.18 ΔF/F; p < 0.0001 v. ATP, one-way ANOVA with Bonferroni’s multiple comparison tests). The P2X_1, 2/3,3_ agonist αβMeATP did not elicit a calcium transient in any of the 107 cells examined. This result suggests that P2X_1,3_ receptors are not readily activated in our imaging experiments and, as such, the Bz-ATP-evoked responses were mediated via P2X_7_ as opposed to P2X_1_ receptors. Punctate P2X_7_ receptor immunoreactivity was observed predominantly in the middle and apical layers of the OE (Figure 1C and D), corroborating the Bz-ATP-induced calcium transients. In our previous study [26] calcium responses were recorded from 14% of the cells following application to P2Y_1,12,13_ agonist ADP, 9% of all cells following application of P2X_3_ agonist β,γ-Methylene-ATP, 2% of all cells following application of P2Y_1,12,13_ agonist 2-methylthioadenosine-5'-O-diphosphate, and no cells following application of AMP or adenosine, both ATP degradation products and adenosine receptor agonists. Collectively, these data using selective agonists suggest the presence of P2Y_2,4,6_ and P2X_1,3,4,7_ receptors that elicit increases in intracellular calcium that could induce the subsequent release of ATP.

### Constitutive and ATP-evoked ATP release occurs in the mouse olfactory epithelium

**Figure 2:**
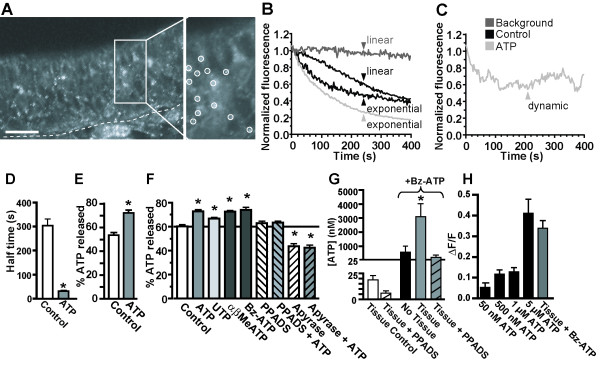
**ATP is released both constitutively and by purinergic receptor activation. (A)**left: Representative confocal image of quinacrine labeling of endogenous ATP stores located ~100 μm into the slice. Dashed line, basement membrane. White box indicates region that is expanded to right. Scale bar, 100 μm. right: Expanded view of OE showing punctate fluorescent ATP stores. White ○’s indicate typical puncta selected for further analysis. **(B-C)** Representative traces of normalized changes in fluorescence over time from individual puncta measured from time-series recordings. (**B**) Shown are background fluorescence measured in tissue devoid of punctate fluorescence (dark gray), and data that display a linear or exponential decrease in fluorescence recorded in control physiological Ringer’s solution (black) or 50 μM exogenous ATP (light gray). (**C**) Representative trace of a punctum displaying dynamic fluctuations in fluorescence recorded following ATP application. Legend pertains to both B and C. **(D)** Half-time decrease in ATP release (mean + SEM) measured from time-series recordings 400 s after application of Control Ringer’s solution (white bar) or 50 μM ATP (grey bar). *, p < 0.0001 v. control (unpaired Student’s t-test). **(E)** Mean (+ SEM) % of released ATP measured from time-series recordings after application of Control Ringer’s solution or 50 μM ATP. **(F)** Mean (+ SEM) % of released ATP measured from Z-stack recordings after application of Control Ringer’s solution, 50 μM ATP, UTP, αβmeATP, or Bz-ATP, or PPADS (25 μM) or apyrase (3 units/ml, 1 hr pre-incubation) ± 50 μM ATP. *, p < 0.01 v. Control (one-way ANOVA with Bonferroni’s planned comparison test). **(G)** Luciferin-luciferase assays were used to quantify the amount of constitutively released ATP or evoked ATP release following stimulation with 50 μM P2X_1,7_ receptor agonist Bz-ATP in the absence or presence of neonatal OE tissue slices. *, p < 0.05 v. Bz-ATP only (one-way ANOVA with Bonferroni’s planned comparison test). **(H)** Peak calcium responses (mean + SEM) elicited from fluo-4 AM loaded P2X_2_-transfected HEK-293 cells following stimulation with ATP in the absence of OE tissue (5 μM, 1 μM, 500 nM or 50 nM) or with 50 μM Bz-ATP in the presence of an OE slice (Tissue + Bz-ATP). Bz-ATP-evoked calcium transients were not observed in transfected HEK-293 cells in the absence of an OE slice (data not shown).

We assessed whether ATP is released in the olfactory epithelium under control physiological conditions and following ATP application. Endogenous ATP stores in neonatal OE slices were labeled with the fluorescent compound quinacrine as previously performed [27]. Quinacrine-labeled ATP stores appear as puncta of fluorescence located throughout the OE (Figure 2A). The individual punctum ranged in diameter between 200 and 250 nm, and sometimes multiple puncta appeared as clusters ranging up to 1.8 μm in diameter, suggesting that quinacrine labels ATP that is stored in large vesicles. We visualized the release of ATP from the fluorescent puncta over a period of 400 seconds under physiological conditions (Ringer’s solution application; Control) and observed either a linear or an exponential decrease in fluorescence intensity (Figure 2B and Table 1). ATP fluorescence in a region of a quinacrine-labeled slice that was devoid of punctate fluorescence (Figure 2B) showed a minimal change in fluorescence over time, suggesting that the decrease in fluorescence of the puncta is not photobleaching of quinacrine, but rather due to ATP release. Application of 50 μM ATP changed the properties of the fluorescent puncta. While some puncta showed an exponential decrease of their fluorescence (Figure 2B, Table 1), fluorescence from other puncta either dynamically fluctuated (Figure 2C, Table 1) or exhibited decreasing fluorescence intensity while the puncta became mobile (Table 1, data not shown). We quantified both the rate of ATP release and the amount of released ATP from all puncta recorded following application of physiological Ringer’s solution (control) or 50 μM ATP. The rate of ATP release was significantly faster following ATP application compared to control (Figure 2D, 31 ± 4 s vs. 304 ± 27 s, n = 11 puncta from 3 slices and 43 puncta from 3 slices, respectively; p < 0.0001, unpaired Student’s t-test). In addition, exogenous ATP (50 μM) significantly increased the amount of released ATP compared to vehicle control (Figure 2E; 65 ± 3% v. 54 ± 2%, p < 0.0001, unpaired Student’s t-test). These data indicate that both constitutive (Ringer’s solution application) and evoked (50 μM ATP application) release of ATP occurs in the mouse olfactory epithelium.

**Table 1:** Characteristics of ATP release from quinacrine-labeled ATP stores*

	**Response**		**Released ATP**	
	**Control**	**ATP**	**Control**	**ATP**
Linear decrease	70% (30/43)	0% (0/61)	51 ±2%	-
Exponential decrease	30% (13/43)	21% (13/61)	59 ±4%	82 ± 3% ^#^
Dynamic fluctuations	0% (0/43)	54% (33/61)	-	68 ±3%
Mobile	0% (0/43)	25% (15/61)	-	45 ±6%

*, Data is presented as mean ± SEM. #, p < 0.001 Student’s t-test.

To confirm that the decrease in fluorescence from the puncta is due to release of quinacrine-labeled ATP rather than moving out of the focal plane, we assessed both constitutive and evoked release of ATP after 400 s using confocal Z stack recordings (see Methods). ATP evoked a significant increase in ATP release compared to control (Figure 2F; 73 ± 1% v. 60 ± 1%; n = 124 puncta from 5 slices and 118 puncta from 5 slices, p < 0.0001, unpaired Student’s t-test). The amount of ATP released from quinacrine-labeled ATP stores under control or ATP-evoked conditions was statistically similar using both the Z stack and the time series recording method (Compare Figure 2E v. 2 F; p > 0.7, unpaired Student’s T tests). These data support the use of Z stack recording to measure both constitutive and ATP-evoked release of ATP. As this method is devoid of putative artifacts related to a shift of focus over time, we used Z stack recordings in all subsequent recordings using quinacrine.

### ATP release is mediated via purinergic receptor activation

We monitored quinacrine-labeled endogenous ATP stores following application of purinergic receptor agonists (50 μM; Figure 2F). Specific P2Y_2,4,6_ agonist UTP (67 ± 1%; n = 173 puncta from 4 slices), P2X_1,2/3,3_ agonist αβMeATP (73 ± 1%; n = 173 puncta from 5 slices), and P2X_1,7_ agonist Bz-ATP (74 ± 2%; n = 29 puncta from 5 slices) significantly increased the amount of released ATP compared to control (60 ± 1%; n = 118 puncta from 5 slices; p < 0.001, one-way ANOVA, Bonferroni’s planned comparisons). The P2Y_2,4,6_ agonist UTP released significantly less ATP than ATP and the selective P2X agonists, αβMeATP and Bz-ATP (p < 0.01, one-way ANOVA, Bonferroni’s planned comparisons). These data suggest that ATP evokes the release of ATP through stimulation of multiple purinergic receptor subunits.

To confirm that ATP-evoked ATP release is linked to stimulation of purinergic receptors, we pre-treated OE slices with either the ectonucleotidase apyrase to degrade extracellular ATP or the non-selective purinergic receptor antagonist PPADS. PPADS pre-treatment (5 min, 25 μM) did not have an effect on the constitutive release of ATP (Figure 2F; 63 ± 2%, n = 157 puncta from 3 slices, p > 0.05 v. control, one-way ANOVA, Bonferroni’s planned comparisons). However, PPADS pre-treatment significantly inhibited the ATP-evoked release of ATP back to control (Figure 2F; 63 ± 1%; n = 152 puncta from 4 slices; p < 0.001 v. ATP, p > 0.05 v. control, one-way ANOVA, Bonferroni’s planned comparisons). Incubation with apyrase (1 hour; 3 units/ml) significantly impaired the constitutive release of ATP compared to control conditions (Figure 2F; 44 ± 2%; n = 90 puncta from 4 slices; p < 0.001, one-way ANOVA, Bonferroni’s planned comparison test). In addition, apyrase pre-treatment (1 hr, 3 units/ml) significantly impaired ATP-evoked release of ATP to levels that were significantly lower than control (Figure 2F; 43 ± 2%, n = 62 puncta from 4 slices; p < 0.001 v. ATP and control, one-way ANOVA, Bonferroni’s planned comparisons). Taken together, our data indicate that ATP evokes release of ATP following purinergic receptor activation.

We next used the luciferin-luciferase assay to quantify the amount of ATP release from neonatal OE slices. Under physiological conditions 19 ± 4 nM ATP was released from OE tissue slices (Figure 2G; n = 8 slices). Although there was a 3-fold decrease in the amount of ATP released in the presence of non-specific purinergic receptor inhibitor PPADS (25 μM), it was not significantly different from control (Figure 2G; 6 ± 2 nM, n = 4 slices, p = 0.07, one-way ANOVA with Bonferroni’s planned comparison). These data are in agreement with the quinacrine-generated data and suggest that constitutive release is not mediated by purinergic receptors. Application of ATP, a co-factor for luciferase, would interfere with the ability to measure endogenously released ATP using the luciferin-luciferase assay. However, the P2X_1,7_ receptor agonist Bz-ATP (50 μM) induced a low level of bioluminescence corresponding to 576 ± 424 nM ATP (Figure 2G; No Tissue (+ Bz-ATP), n = 5 slices), allowing the quantification of evoked ATP-release. Bz-ATP (50 μM) evoked a significant 5-fold increase in bioluminescence in the presence of OE tissue slices (Figure 2G; Tissue (+ Bz-ATP): 3108 ± 914 nM ATP, n = 7 slices, p < 0.05 v. no tissue, one-way ANOVA with planned comparisons). Significantly more ATP was released from slices stimulated with 50 μM Bz-ATP than from control conditions (Figure 2G; p < 0.01; one-way ANOVA with planned comparisons). The non-specific purinergic receptor inhibitor PPADS (25 μM) significantly inhibited the Bz-ATP-evoked release of ATP to control (no tissue) levels (Figure 2G; 215 ± 150; n = 4 slices; p > 0.05, one-way ANOVA with planned comparisons). These data confirm the involvement of P2X_1,7_ receptors in the evoked release of ATP.

We next used stable P2X_2_-transfected HEK-293 cells as biosensors to further confirm that ATP is released from OE slices. Fluo-4 AM loaded P2X_2_-transfected HEK-293 cells were stimulated with ATP concentrations ranging from 20 nM, the concentration of ATP constitutively released from OE slices as measured by the luciferin-luciferase assay, to 5 μM, the concentration released from OE slices by Bz-ATP stimulation. ATP application (20 nM) was insufficient to elicit an increase in intracellular calcium (data not shown) and 50 nM ATP induced a very small calcium response in fluo-4 AM loaded P2X_2_-transfected HEK-293 cells (Figure 2H, 0.05 ± 0.02 ΔF/F, n = 10 cells). The mean peak (± SEM) ATP response elicited by 5 μM (0.41 ± 0.07 ΔF/F, n = 15 cells) was significantly higher than the mean peak amplitudes elicited by all lower ATP concentrations (1 μM: 0.13 ± 0.02 ΔF/F, n = 13 cells; 500 nM: 0.12 ± 0.02 ΔF/F, n = 19 cells, p < 0.05, one-way ANOVA with Bonferroni’s planned comparison tests). Pretreatment with PPADS (25 μM) totally abolished the ATP-induced calcium transients in the P2X_2_-transfected HEK-293 cells (0.01 ± 0.01 ΔF/F, n = 10 cells from 3 coverslips; data not shown), indicating the calcium increase was mediated by activated P2X_2_ receptors. Neither control Ringer’s solution nor Bz-ATP (50 μM) induced increases in intracellular calcium in the P2X_2_-transfected HEK-293 cells (data not shown). These data suggest that P2X_2_-transfected HEK-293 cells are not sensitive enough to detect constitutively released ATP (~20 nM, Figure 2F). Indeed, no calcium increases were detected in P2X_2_-transfected HEK-293 cells located adjacent to an OE slice in the recording chamber (n = 3 coverslips; data not shown). However, the P2X_2_-transfected HEK-293 cells are sensitive enough to detect the evoked release of ATP from OE slices which is ~3 μM (see Figure 2G). In the presence of an OE slice, 50 μM Bz-ATP induced a mean (± SEM) calcium transient peak amplitude of 0.34 ± 0.04 ΔF/F in the P2X_2_-transfected HEK-293 cells (Figure 2H, n = 70 cells from 3 coverslips). As Bz-ATP did not elicit calcium increases in P2X_2_-transfected HEK-293 cells in the absence of OE tissue, these data indicate that Bz-ATP evokes the release of ATP from OE slices. Note that the calcium response elicited by Bz-ATP stimulation of an OE slice was similar to that elicited by 5 μM ATP in the absence of an OE slice (Figure 2H; p > 0.05, one-way ANOVA with Bonferroni’s planned comparisons). This result suggests that approximately 5 μM ATP is released from a slice following stimulation with Bz-ATP and is consistent with the data obtained by the luciferin-luciferase assays. Taken together, data obtained from fluo-4 AM loaded P2X_2_-transfected HEK-293 cells show that ATP is released from OE slices following P2X_1,7_ purinergic receptor stimulation.

### Mechanisms of ATP-evoked ATP release

**Figure 3:**
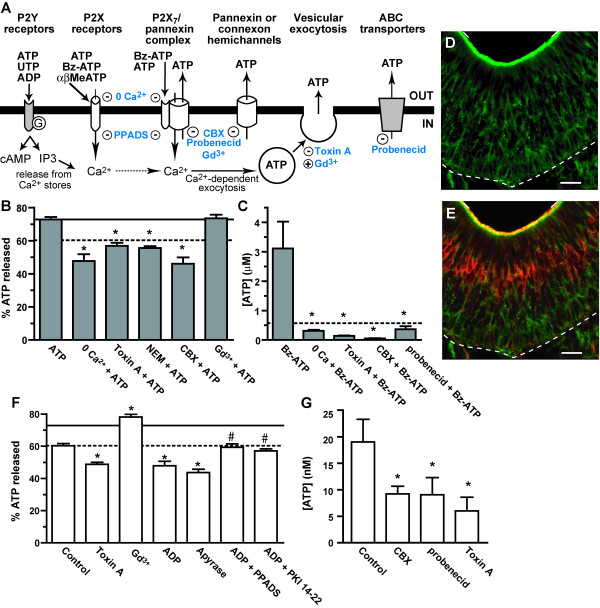
**Mechanisms of ATP release. (A)** Schematic of the possible mechanisms of ATP release investigated in this study. Extracellular ATP, leaking from damaged cells or released constitutively, activates both P2Y and P2X purinergic receptors, leading to increases in intracellular Ca^2+^ and Ca^2+^-dependent exocytosis. ATP activated P2X_7_ receptors may form a complex with pannexin hemichannels through which ATP can efflux. ATP release may also occur through pannexin/connexin hemichannels or ATP binding cassette (ABC) transporters. Pharmacological inhibitors (−) and stimulators (+) are indicated in blue. **(B-C)** Measurement of agonist-evoked ATP release. (B) Mean (+ SEM) % of released ATP measured from Z-stack recordings after application of ATP (50 μM) following incubation in Ringer’s solution (ATP), media lacking extracellular calcium (0 Ca^2+^ + ATP), non-specific pannexin and activated P2X_7_ receptor inhibitor carbenoxolone (100 μM, CBX + ATP), vesicle fusion inhibitors *Clostridium difficile* toxin A (30 min,1 nM, Toxin A + ATP) and N-ethylmaleimide (500 μM, NEM + ATP), or vesicular release stimulator and pannexin and connexin hemichannel inhibitor gadolinium chloride (50 μM, Gd^3+^ + ATP). *, p < 0.001 v. ATP, one-way ANOVA with Bonferroni’s planned comparison test. Mean ATP value indicated by solid line; mean control value (see Figure 2F) indicated by dashed line. (C) Luciferin-luciferase assays were used to quantify the amount of ATP released (mean + SEM) following application of 50 μM P2X_1,7_ receptor agonist Bz-ATP in calcium-free solution (0 Ca^2+^ + Bz-ATP), carbenoxolone (100 μM; CBX + Bz-ATP), selective pannexin 1 channel and ABC transporter inhibitor probenecid (500 μM), or *Clostridium difficile* toxin A (30 min, 1 nM; Toxin A + Bz-ATP). *, p < 0.01 v. Bz-ATP, one-way ANOVA with Bonferroni’s planned comparison test. Dashed line indicates the mean concentration of ATP measured when 50 μM Bz-ATP was used in the assay in the absence of tissue. **(D-E)** Immunoreactivity of synaptosomal associated protein SNAP-23 (green; D,E) and olfactory marker protein, a marker for neurons (red; E). Dashed line, basal lamina; solid line, apical surface of OE. Scale bar: 20 μm. **(F-G)** Measurement of constitutive ATP release. Confocal Z-stack imaging of quinacrine fluorescence (F) and luciferin-luciferase assays (G) were performed to quantify constitutive ATP release following incubation with Ringer’s solution (Control), or various inhibitors: *Clostridium difficile* toxin A (30 min; 1 nM; Toxin A), gadolinium chloride (50 μM; Gd^3+^), probenecid (500 μM), carbenoxolone (100 μM; CBX), ectonucleotidase apyrase (1 h; 3 units/ml), P2Y_1,6,12,13_ agonist ADP (50 μM), non-selective purinergic receptor antagonist PPADS and ADP (5 min; 25 μM and 50 μM, respectively), or PKA inhibitor protein kinase inhibitor fragment 14–22 (PKI 14–22) and ADP (1 hour; 10 μM and 50 μM, respectively). Data reported as described above in Figure 3 B-C. *, p < 0.05 v. Control; #, p < 0.001 v. ADP, one-way ANOVA with Bonferroni’s planned comparison test.

We tested the hypothesis that ATP-evoked ATP release in the olfactory epithelium is similar to that in other organ systems and investigated the roles of purinergic receptors, vesicular exocytosis, pannexin/connexin hemi channels and ABC transporters (see Figure 3A for model). Quinacrine imaging was used to investigate vesicular exocytosis as this technique allows visualization of vesicular ATP release, and luminometry assays were used to quantify ATP release for all the proposed mechanisms. Our data support the hypothesis that ATP-evoked release of ATP is mediated predominantly by ionotropic P2X_1,3,7_ receptors (Figure 2F-H), but there is evidence that G-protein coupled P2Y_2,4,6_ receptors may also be involved (Figure 2F). Activation of either receptor subtype increases intracellular calcium which could induce calcium-dependent exocytosis. Removal of extracellular calcium significantly inhibited ATP-evoked release of ATP (Figure 3B-C; 48 ± 4%, n = 30 puncta from 4 slices; 315 ± 34 nM, n = 7 slices; p < 0.05 v. ATP or Bz-ATP, Student’s t-test). These data further support the involvement of ionotropic P2X_1,3,7_ receptors, but does not rule out the possibility of a P2Y_2,4,6_ receptor contribution. We hypothesized that increases in intracellular calcium following purinergic receptor activation may lead to calcium-dependent exocytosis of ATP stored in vesicles. Pre-treatment with *Clostridium difficile* toxin A (15 min, 0.5 nM), known to impair the fusion of vesicles and potently inhibit exocytosis, significantly impaired the evoked release of ATP (Figure 3B-C; 57 ± 2%, n = 91 puncta from 3 slices; 138 ± 16 nM ATP; n = 3 slices; p < 0.01 v. ATP or Bz-ATP, one-way ANOVA with Bonferroni’s planned comparison tests). In addition, *N*-ethylmaleimide, another vesicle fusion inhibitor, significantly inhibited the ATP-evoked release of ATP (Figure 3B; 56 ± 1%, n = 226 puncta from 3 slices, p < 0.001 v. ATP, one-way ANOVA with Bonferroni’s planned comparison test). Exocytosis requires formation of soluble *N*-ethylmaleimide-sensitive factor attachment protein receptor (SNARE) core complexes [28]. We examined the expression of the SNARE complex protein synaptosomal associated protein (SNAP)-23. SNAP-23 localized to the thin cytoplasmic extensions throughout the OE that could be either sustentacular cells or neurons (Figure 3D-E). Taken together, our data indicate that purinergic receptor-mediated calcium-dependent exocytosis evokes the release of ATP.

ATP can also be released through a large non-selective pore formed either by the P2X_7_ receptor [29] or by the P2X_7_ receptor in a complex with pannexin [30]. To test if ATP or Bz-ATP evokes the release of ATP via a P2X_7_-pannexin complex, we used carbenoxolone (100 μM), a non-specific pannexin and activated P2X_7_ receptor inhibitor [31,32]. Carbenoxolone significantly decreased the amount of ATP released (Figure 3B-C; 46 ± 4%, n = 34 puncta from 3 slices; 56 ± 9 nM, n = 3 slices; p < 0.01 v. ATP or Bz ATP, one-way ANOVA with Bonferroni’s planned comparisons). In addition, probenecid (500 μM), a selective pannexin 1 channel inhibitor [31,33] as well as an ABC transporter inhibitor [17], significantly decreased the amount of ATP released from slices stimulated by Bz-ATP measured by luminometry (Figure 3C; 365 ± 106 nM, n = 4 slices; p < 0.01, one-way ANOVA with Bonferroni’s planned comparisons). These data indicate that the P2X_7_ receptor and pannexin 1 channels, alone or in a complex, and ABC transporters may also be involved in ATP-induced ATP release.

### Mechanisms of constitutive ATP release

Our data indicated that purinergic receptors are not involved in the constitutive release of ATP based on the lack of an effect on ATP release in the presence of purinergic receptor antagonist PPADS (Figure 2E). Thus, we tested the hypothesis that constitutive ATP release in the olfactory epithelium is similar to that in other organ systems and investigated the role of pannexin/connexin hemi-channels, ABC transporters and vesicular exocytosis (see Figure 3A for models) using both luminometry assays and quinacrine imaging. We measured ATP release in the presence of the non-specific pannexin inhibitor carbenoxolone (100 μM). Carbenoxolone decreased the amount of constitutive ATP released by half (Figure 3G, 9 ± 1 nM, n = 5 slices, p < 0.05 v. control, one-way ANOVA with Bonferroni’s planned comparison). In addition, the selective pannexin 1 and ABC transporter inhibitor probenecid (500 μM) significantly decreased the amount of ATP released from OE slices (Figure 3G; 9 ± 3 nM ATP, n = 4 slices; p < 0.05 v. control, one-way ANOVA with Bonferroni’s planned comparison). Use of vesicular fusion inhibitor *Clostridium difficile* toxin A (1 nM) significantly impaired the release of ATP compared to control conditions (Figure 3F; 49 ± 1%, n = 329 puncta from 3 slices, p < 0.001, 6 ± 3 nM ATP; n = 3 slices; p < 0.05, one-way ANOVA with Bonferroni’s planned comparisons). We also used gadolinium chloride (Gd^3+^, 50 μM) that enhances spontaneous exocytosis in rat brain synaptosomes [34] as well as inhibits pannexin and connexin hemichannels [35]. Gd^3+^ significantly increased constitutive exocytotic release of ATP (Figure 3F; 78 ± 2%, n = 75 puncta from 3 slices; p < 0.001, one-way ANOVA with Bonferroni’s planned comparisons). Note that in the presence of Gd^3+^, ATP did not significantly enhance the release of ATP compared to Gd^3+^ alone or ATP alone (Figure 3B, 74 ± 2% ATP, n = 49 puncta from 3 slices, p > 0.05 v. ATP and v. Gd^3+^, one-way ANOVA with Bonferroni’s planned comparisons). These results suggest that exocytotic release of ATP may have a greater role in the constitutive release of ATP than release through pannexin/connexin hemichannels. In addition, Gd^3+^-evoked vesicular release of ATP may be operating at the maximum extent measurable under physiological conditions as ATP did not enhance the release of ATP in the presence of Gd^3+^. Alternatively, it may not be possible to observe a greater release of ATP under the quinacrine experimental conditions, and it is not possible to measure the effects using luminometry as the trivalent cation interferes with the luciferin-luciferase assay. Overall, our data show that a constitutive release of ATP involves spontaneous exocytosis, ABC transporters and pannexins in neonatal mouse OE.

We observed that degradation of extracellular ATP by apyrase inhibited the release of ATP to levels even lower than control (Figure 2E). In mouse neuromuscular junction the spontaneous release of neurotransmitters is inhibited by stimulation of the G protein-coupled P2Y purinergic receptors [36]. We hypothesized that in the presence of apyrase, the constitutively released ATP could be degraded to ADP that could inhibit the spontaneous release of ATP via P2Y_1,12,13_ purinergic receptor activation. Application of ADP (50 μM) significantly impaired the release of ATP (Figure 3F; 48 ±3% ATP, n = 80 puncta from 3 slices; p < 0.0001 v. control, unpaired Student’s t-test). This result confirms that activation of P2Y_1,12,13_ receptor subtypes inhibits the spontaneous vesicular release of ATP. Therefore, blockade of the P2Y_1,6,13_ receptors with PPADS should prevent the inhibitory effect of ADP. As expected, following PPADS treatment (5 min; 25 μM), the amount of released ATP after ADP stimulation increased significantly and was back to control level (Figure 3F; 59 ± 2%; n = 78 puncta from 3 slices; p < 0.001 vs. ADP and p > 0.05 v. control, one-way ANOVA, Bonferroni’s planned comparison test). Activation of P2Y_1,12,13_ receptors by ADP can either activate or inhibit PKA via adenylyl cyclase-mediated cAMP production. We therefore hypothesized that PKA might be involved in the ADP-induced inhibition of spontaneous ATP exocytosis. If ADP inhibits PKA, we would expect that PKA inhibition would have the same effect on spontaneous ATP exocytosis as ADP application alone. On the other hand, if ADP activates PKA, the PKA inhibitor should increase the amount of released ATP compared to ADP alone. Incubation with protein kinase inhibitor fragment 14–22 (1 hr, 10 μM) prevented ADP-mediated inhibition of the spontaneous ATP exocytosis (Figure 3F; 57 ± 1%; n = 130 puncta from 3 slices; p < 0.001 v. ADP and p > 0.05 v. control, one-way ANOVA, Bonferroni’s planned comparison test). Taken together, these data suggest that stimulation of P2Y_1,13_ receptor subtypes that lead to PKA activation down-regulates spontaneous exocytosis of ATP occurring during the constitutive ATP release in mouse neonate OE.

### Constitutive ATP release induces proliferation

**Figure 4:**
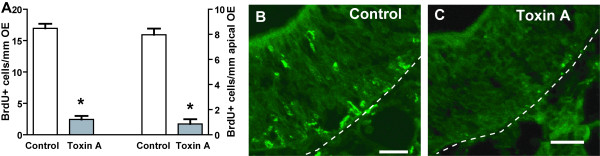
**Constitutive exocytotic release induces proliferation in the mouse OE. (A)** Quantification of the number of BrdU-immunoreactive cells in the mouse OE and in the apical region of olfactory epithelium (apical OE) 3 days following intranasal instillation with saline (Control) or Clostridium difficile toxin A (Toxin A; 1 nM). *, p < 0.001 v. control using one-way ANOVA with Bonferroni’s planned comparison test. n = 15 and 17 sections from 3 and 4 animals for control and toxin A, respectively. **(B-C)** Representative BrdU immunoreactivity (green) in OE turbinates exposed to saline control (B) or Toxin A (C). Apical region is oriented to the top and the dashed white line represents the basement membrane. Scale bar is 20 μm.

We previously observed that ATP induces neuroproliferation via purinergic receptor activation [9,10]. To determine if constitutive ATP release induces neuroproliferation, we examined the effect of *Clostridium difficile* toxin A, an inhibitor of constitutive release of ATP on cell proliferation in the OE. Neonatal mice were intranasally instilled with either saline vehicle or 1 or 10 nM toxin A. Three days post-instillation, mice were injected with BrdU and 6 h later tissue was collected to visualize proliferating cells. *Clostridium difficile* toxin A significantly decreased BrdU incorporation throughout the OE and in the apical region of the OE compared to vehicle control (Figure 4: 2.4 ± 0.6 v. 17.0 ± 0.7 BrdU^+^ cells/mm OE and 0.8 ± 0.4 v. 8.0 ± 0.5 BrdU^+^ cells/mm apical OE; p < 0.0001, unpaired Student’s t-test). While we cannot rule out that the toxin might damage the OE, we would expect that cell damage or cell death caused by exposure to Clostridium difficile toxin A should increase BrdU incorporation because of post-injury neuroregeneration. Thus, taken together, this data suggests that constitutive, toxin A sensitive-exocytosis of ATP or another substance induces increased proliferation and may have a role in the normal turnover of both the olfactory sensory neurons and the sustentacular cells.

## Discussion

In this study, we demonstrated the presence of ATP-evoked ATP release in neonatal mouse OE slices, and determined the mechanisms underlying this phenomenon. We show that activation of purinergic receptors increases intracellular calcium which could subsequently induce calcium-dependent exocytosis of ATP. Although multiple P2Y and P2X selective purinergic receptor agonists triggered release of ATP, our data suggest that P2X_1,7_ receptor subtypes play key roles. Indeed, the selective P2X_1,7_ agonist BzATP significantly increases the exocytotic release of ATP. Only small cations are permeable through the P2X_7_ receptor under physiological conditions. However, in the maintained presence of high concentrations of ATP, when divalent cation levels are low, the P2X_7_ receptor can either form a non-selective pore [29], or, after complexing with pannexin, form a large pore through which ATP can flow [30]. Bz-ATP is maintained for 30s - 10 min in these studies, suggesting ample time to open the large pore of the P2X_7_ receptor.

In the OE, ATP has been ascribed the functions of neuromodulation, neuroprotection and neuroproliferation by our laboratory and other research groups [9,10,12,13,26,37,38]. The evoked release of ATP could be triggered pathologically by the presence of increased extracellular ATP levels because of ATP leaking from damaged cells. Indeed, toxicant exposure depletes intracellular ATP [39], presumably due to released ATP from damaged cells [37]. The evoked release of ATP could play a role in any of these functions. The sustained release of ATP could either induce the neuroprotective expression of heat shock proteins [37], directly stimulate the progenitor cells to proliferate [9,38] and differentiate [9], or act indirectly by promoting the release of other neurotrophic factors [14]. Thus, the results in this study identify the release mechanisms involved in ATP-mediated post-injury neuroregeneration.

We previously showed indirectly that constitutive release of ATP might modulate odorant responsiveness of the olfactory sensory neurons [26]. Here, using luciferin-luciferase assays, we directly confirm that constitutive release of ATP occurs via exocytosis and efflux through ABC transporters. We also demonstrate a negative feedback system for constitutive ATP release regulated by ADP, P2Y receptor stimulation and subsequent activation of PKA. The exact mechanism of constitutive ATP release inhibition is still unknown. To our knowledge, direct inhibition of exocytosis or ABC transporters does not involve PKA. P2Y receptor activation inhibits spontaneous vesicular release of acetylcholine in the mouse neuromuscular junction, but does not require PKA [36,40]. ADP is rapidly degraded to adenosine. Our previous work suggests that adenosine receptors are not expressed in the olfactory epithelium: we observed no responses to adenosine or AMP during confocal calcium recordings [26]. However, adenosine may modulate ADP’s activation of P2Y receptors to regulate constitutive ATP release.

We also postulate that constitutively released ATP might play a role in neuronal homeostasis in the OE. Purinergic receptor activation induces cell proliferation [9], has post-injury proliferative and protective functions [10], and elicits upregulation and/or release of many different neurotrophic factors [12-14]. Here, we show constitutive exocytosis may induce cell proliferation. Neuronal homeostasis must be tightly regulated to prevent over-stimulation of cell proliferation. The P2Y receptor-mediated inhibition of the constitutive release of ATP would provide negative regulation. Further support for this negative feedback system is provided by the observation that basal progenitor cells of the OE express ectonucleotidases [41]. This provides a mechanism to degrade ATP to ADP, preventing excessive stimulation of P2Y_2_ receptors present on the proliferative basal progenitor cells [26], and inhibiting the constitutive release of ATP. Thus, there is local regulation of constitutive ATP release.

## Conclusions

We describe constitutive and purinergic receptor-mediated ATP-evoked ATP release via calcium-dependent vesicular exocytosis, pannexin/connexin hemichannels and ABC transporters. Overall, the demonstration that extracellular ATP can elicit ATP release provides a novel pathway with potential pharmacological targets to explore mechanisms of neuroregeneration and that have ramifications for the CNS. Likewise, the role of constitutive release of ATP in neurogenesis under physiological conditions may be utilized to promote neuroregeneration following cell injury in neurodegenerative disease and spinal cord injury.

## Methods

### Solutions

All chemicals were purchased from Sigma-Aldrich (St. Louis, MO, USA) unless noted.

Ringer’s solution contained (in mM) 140 NaCl, 5 KCl, 1 MgCl_2_ 6H_2_O, 2 CaCl_2_, 10 HEPES, 10 glucose (pH 7.4). Calcium-free Ringer’s solution contained (in mM) 140 NaCl, 5 KCl, 10 HEPES, 4 EGTA, and 20 glucose (pH 7.4). All solutions used in the quinacrine experiments contained probenecid (500 μM), an anionic transporter inhibitor, to aid in retention of quinacrine [42]. Ethanol and dimethylsulfoxide interfered with both the quinacrine and the luciferin-luciferase assays, and thus only inhibitors that soluble in Ringer’s solution were used in these studies. Concentrated stock solutions of ATP, UTP, cytidine 5’-triphosphate (CTP), 2’(3’)-O-(4-benzoylbenzoyl)adenosine 5’-triphosphate (Bz-ATP), α,β-methylene adenosine 5’-triphosphate (αβMeATP), pyridoxal-phosphate-6-azophenyl-2′,4′-disulfonate (PPADS), apyrase, N-ethylmaleimide, and *Clostridium difficile* toxin A were made in Ringer’s solution, stored at −20°C and, on the day of the experiment, diluted to concentration values indicated in the text. Fresh stock solution of carbenoxolone dissolved in Ringer’s solution was made on the day of the experiment and diluted to concentration values indicated in the text.

### Cell and tissue and culture

Olfactory epithelial slices (400–500 μm) were prepared from Swiss Webster neonatal mice (Charles River, Portage, MI) as previously described [26]. All animal procedures were approved by Michigan State University’s IACUC, and all applicable guidelines from NIH were followed. P2X_2_-transfected human embryonic kidney (HEK-293) cells were generously provided by Dr. James Galligan from Michigan State University [43]. They were grown in Dulbecco’s modified Eagle’s medium (DMEM) F-12 containing 10% fetal bovine serum, 10% GluMax (Invitrogen, Carlsbad, CA, USA), and 100 units mL^−1^ penicillin and streptomycin. Cells were passaged once every 3 days when they reached 90% confluence. Afterwards, cells were plated on 35 mm coverslips and maintained at 37°C (5% CO_2_) for 24 h before use in calcium imaging experiments. For each treatment group 3 coverslips were used.

### Bioluminescence detection of ATP released from olfactory epithelium slices

ATP was quantified using 0.5 mM d-luciferin and 4 μg/ml luciferase in Ringer's solution obtained from an ATP determination kit (Molecular Probes, Eugene, OR). Luminescence, in relative light units, was recorded continuously with 1-s photon collection intervals using a Turner TD20/20^n^ luminometer. Standard curves of ATP (Mg salt) ranging between 10 nM - 5 μM and made using serial dilution from a 0.5 M ATP stock were recorded daily in 200 μl luciferin and luciferase solution. The ATP standard curves were linear in the range of 10 nM - 5 μM (r^2^: 0.93–0.99). Individual OE slices (500 μm) were placed in a 200 μl bolus of luciferin and luciferase solution in a 35 mm petri dish and ATP release rates measured in presence or absence of inhibitors. In order to measure purinergic-evoked ATP release using the luciferin-luciferase assay, the purinergic agonist should not act as a substrate or co-factor for luciferase. We tested the ability of Bz-ATP, UTP and CTP to produce luminescence in the luciferin-luciferase assay. Bz-ATP (50 μM) induced the smallest increase in luminescence when applied alone to the luciferin and luciferase solution, corresponding to ~500 nM ATP, whereas UTP and CTP induced a response that corresponded to 5 μM ATP. In pilot studies, the luminescence baseline was stabilized at t = 10 minutes after a slice or Bz-ATP was put into the luciferin and luciferase solution. Therefore, all data were analyzed at 10 minutes by converting the raw relative light unit values to concentration of ATP using the standard curve (relative light units v. ATP concentration (μM)) and interpolation from a linear regression (Prism Software 5.01, Graphpad Software; San Diego, CA). Note that the estimated concentration of released ATP (into 200 μl) is likely to be an underestimate of the amount released into the microenvironment of the tissue. At the end of each experiment, as a positive control, a bolus of Triton X-100 was added (0.5% final concentration) to release ATP via lysis of the cells.

### Confocal imaging of fluorescent ATP stores

OE slices were incubated for 30 min at room temperature (23°C) in Ringer’s solution with quinacrine (5 μM). Note the diameter of fluorescent punctum (~200–250 nm) that resulted from quinacrine loading was within the optical resolution of confocal fluorescent microscopy. Recordings were performed at a depth of at least 100 μm to avoid damage using an Olympus Fluoview 1000 system (Olympus, Center Valley, PA) [44] and a superfusion flow rate of 1.0 ml/min. A static bath in which agonist or inhibitor were added directly to the bath was used in a few cases due to excessive movement of the slice (n = 6 slices). No statistical differences were observed between results recorded in a static or superfused bath (Table 2). Therefore, additionally, to minimize the application of select inhibitors in limited supply, experiments using the ectonucleotidase apyrase and *Clostridium difficile* toxin A were performed in a static bath. Quinacrine was excited at 488 nm and fluorescence was detected at 510–525 nm. Images ranged from 512 × 512 pixels to 1600 × 1600 pixels.

**Table 2:** Comparison of ATP release in different experimental conditions*

**Treatment**	**Superfused bath**		**Static bath**		**Significance (Student’s t test)**
	**n**	Released ATP*	**n**	Released ATP*	
Control (Ringer’s)	89 puncta, 3 slices	65 ± 1%	80 puncta, 3 slices	66 ± 2%	*p* = 0.61
PPADS	75 puncta, 2 slices	62 ± 3%	82 puncta, 2 slices	64 ± 1%	*p* = 0.41
αβMeATP	144 puncta, 4 slices	73 ± 1%	29 puncta, 1 slice	70 ± 1%	*p* = 0.09

*, Data is presented as mean ± SEM.

Time series experiments were performed at ≥ 1 Hz and data were collected for 400 s, the time required for the fluorescence decrease of most of the puncta recorded in control condition (Ringer’s application) to reach a plateau. For each punctum, the percentage of released ATP was determined as 100*[1-(F/F_0_)], where F_0_ is the mean fluorescence of the first 10 frames and F is the fluorescence remaining after 400 s. We also calculated the half-time of ATP fluorescence by fitting the change in fluorescence over time with a non-linear regression function (exponential decay) or linear regression function (linear decay). We analyzed only puncta with linear or exponential fluorescence due to the irregularity of the dynamic and mobile puncta.

Z-stack recordings were also performed to minimize changes in the fluorescence of quinacrine-labeled ATP inherent with imaging thick tissue. We recorded for a duration of 500 s by collecting 5 consecutive Z-stacks per slice, each with a duration of 100 s. Ringer’s solution or pharmacological compounds were applied at the beginning of the second Z-stack (i.e., at 100 s). In Z-stack recordings, only one focal plane was selected for further analysis based on the ability to distinguish the same cell morphology in relationship to the same puncta of fluorescence within the one focal plane throughout the 500 s recording. For each punctum, the percentage of released ATP was determined as 100*[1-(F/F_0_)], where F_0_ is the fluorescence of the punctum in the focal plane of the first Z stack (i.e., t = 0–100 s, depending on the focal plane selected) and F is the fluorescence remaining in the punctum of the same focal plane of the last Z stack, (i.e., t = 400–500 s, depending on the focal plane selected). Note the Z-stack F value is measured ~400 s following agonist application to correspond as closely as possible to the time-series experiments. Baseline fluorescence intensity did not vary between slices immediately following quinacrine loading and following long incubations with control Ringer’s solution or inhibitors. Therefore, there were no adjustments made to accommodate incubation time. For each time-series and Z-stack experiment 22 to 321 puncta from at least 3 slices were analyzed.

### Confocal calcium imaging

Live cell confocal imaging was performed as previously described on olfactory epithelial slices (400–500 μm) [26] and/or P2X_2_-transfected HEK 293 cells (n = at least 3 slices/coverslips) using an Olympus Fluoview 1000 system. OE slices and P2X_2_-transfected HEK 293 cells were loaded with fluo-4 AM (18 uM; Invitrogen, Carlsbad, CA, USA) for 90 and 30 minutes, respectively, as previously described [26]. Time series experiments were performed collecting 256 × 128 pixel images at 0.7 Hz for slices and ≥ 1 Hz for HEK-293 cells. HEK-293 cells were recorded for 10 minutes as pilot experiments showed that these cells always responded to ATP application within this time frame. When using OE slices, experiments were performed by sequentially superfusing purinergic receptor agonists in a randomized order that differed from slice to slice. The fluorometric signals obtained are expressed as relative fluorescence (F) change, ΔF/F = (F-F_0_)/F_0_, where F_0_ is the basal fluorescence level (mean F of first 10 frames). Increases in fluorescence 10% above baseline fluorescence fluctuations were considered responses. Only cells that responded to a final ATP application were included in the data analysis and only responses were included in the analysis for peak height determination.

### In vivo study

Neonatal mice (post-natal day 1) were instilled intranasally with 10 μl saline solution ± *Clostridium difficile* toxin A (1 nM). Each mouse received a re-instillation of saline ± *Clostridium difficile* toxin A every 24 hrs. Mice received an intraperitoneal injection of bromodeoxyuridine (BrdU; 180 mg/kg total) at 66, 68 and 70 h and tissue was collected at 72 hrs by decapitation. The retronasal passage was flushed with 4% paraformaldehyde (PFA) in 0.1 M phosphate-buffered saline (PBS) and tissue was stored in 4% PFA at 4°C overnight. Tissue was rinsed in 0.1 M PBS, cryoprotected in a 20% sucrose solution at 4°C overnight, placed in dry ice for 10 min, wrapped in parafilm and foil, and stored at −80°C until sectioning. Cryostat sections (20 μm) were collected on superfrost plus slides (Electron Microscopy Sciences, Hatfield, PA), and stored at −20°C.

### Immunohistochemistry

To perform BrdU immunohistochemistry, tissue sections were warmed to room temperature, rehydrated with 0.1 M PBS, permeabilized with 0.3% Triton-X in 0.1 M PBS and then blocked with 10% normal donkey serum (NDS) diluted in 0.1 M PBS for 1 h at room temperature. To denature DNA, sections were incubated in filtered 2 M HCl for 30 min at 65°C. Tissue was treated with rat anti-BrdU monoclonal primary antibody (1:100 in 10% NDS; Abcam, Cambridge MA) at 4°C overnight. Tissue sections were treated with donkey anti-rat FITC secondary antibody (1:200 in 0.3% Triton X-100; Jackson ImmunoResearch Laboratories, West Grove, PA) for 2 hrs at 37°C, rinsed with 0.1 M PBS and mounted with Vectashield (Vector Laboratories, Burlingame, CA). A Nikon Eclipse 2000-U microscope equipped with an xcite 120™ fluorescence illumination system was used to visualize immunoreactivity. The total number of BrdU^+^ cells was counted over a measured length of the olfactory epithelium lining the turbinates of each section. BrdU^+^ cells found in the apical region of the turbinates were also counted. n = 15, 17, 19 sections from 3 and 4 animals for control and 1 nM toxin A, respectively.

All other immunohistochemistry was performed by rehydrating paraformaldehyde-fixed tissue sections from adults (20 μm) collected as previously described [9] with 0.1 M PBS with 0.1% triton X-100 for 20 min, and blocked with bovine serum albumin (BSA) (3%, P2X7; 5%, SNAP 23) in 0.1 M PBS with triton X-100 for 60 min. Primary antibodies were added to tissue sections overnight. Goat-anti P2X_7_ antibody (1:20; Santa Cruz, CA) and rabbit-anti SNAP 23 + goat-anti olfactory marker protein (OMP)(1:200; Abcam, Cambridge, MA and 1:250, Waco Chemicals, Plano, TX, respectively) antibodies were made in 1% BSA in 0.1 M PBS. FITC-conjugated donkey anti-rabbit or donkey anti-goat antibody (1:200; Jackson ImmunoResearch Labs, West Grove, PA) was applied for 1 hr at room temperature. Sections were then washed and mounted in Vectashield mounting medium for fluorescence (Vector Labs, Burlingame, CA) and visualized on an Olympus FluoView 1000 laser scanning confocal microscope. FITC dye was excited at 488 nm and low pass filtered at 505–525 nm. Antibody specificity was tested by omitting the primary antibodies. Additionally, a peptide neutralization protocol was performed by combining P2X_7_ antibody (0.04 mg/ml) with a 10–20 fold excess of the immunizing peptide (0.4-0.8 mg/ml). No immunoreactivity was observed in any of the controls.

### Statistical analysis

We employed either the Student’s t-test or a one-way ANOVA followed by Bonferroni’s planned comparison test without correction for multiple comparisons to compare data sets, as indicated in text, using Prism Software 5.01 (Graphpad Software; San Diego, CA). A value of *p* < 0.05 was considered statistically significant.

## Competing interests

The authors declare that they have no competing interests.

## Author’s contributions

SH participated in the experimental design, carried out all the ATP release experiments, the confocal calcium imaging study using HEK-293 cells and the in vivo proliferation study, and drafted the manuscript. CJ carried out the immunohistochemistry. CCH conceived of the study, participated in its design and coordination, carried out the confocal calcium imaging study using neonatal OE slices, and wrote the manuscript. All authors read and approved the final manuscript.
